# Uncovering the Interaction between TRAF1 and MAVS in the RIG-I Pathway to Enhance the Upregulation of IRF1/ISG15 during Classical Swine Fever Virus Infection

**DOI:** 10.3390/cells13131165

**Published:** 2024-07-08

**Authors:** Liyuan Zhang, Rongze Tang, Dongli Liang, Wenfeng Wang, Kaijun Min, Tingrong Luo, Xiaoning Li

**Affiliations:** 1 College of Animal Sciences and Veterinary Medicine, Guangxi University, Nanning 530004, China; 2118402003@st.gxu.edu.cn (L.Z.); 2018393002@st.gxu.edu.cn (R.T.); 2218302019@st.gxu.edu.cn (D.L.); 2018302034@st.gxu.edu.cn (W.W.); 2218393043@st.gxu.edu.cn (K.M.); 2 State Key Laboratory for Conservation and Utilization of Subtropical Agro-Bioresources, Guangxi University, Nanning 530004, China; 3 Guaxi Zhuang Autonomous Region Engineering Research Center of Veterinary Biologics, Nanning 530004, China; 4 Guangxi Key Laboratory of Animal Breeding, Disease Control and Prevention, Nanning 530004, China

**Keywords:** CSFV (classical swine fever virus), TRAF1 (tumor necrosis factor receptor (TNF-R)-associated factor 1), RIG-I (retinoic acid-inducible gene I), MAVS (mitochondrial antiviral-signaling protein), IRF1 (interferon regulatory factor 1), ISG15 (interferon-stimulating gene 15)

## Abstract

Classical swine fever (CSF) is caused by the classical swine fever virus (CSFV), which poses a threat to swine production. The activation of host innate immunity through linker proteins such as tumor necrosis factor receptor (TNF-R)-associated factor (TRAF) is crucial for the induction of the NF-κB pathway. Recent research has revealed the involvement of mitochondrial antiviral-signaling protein (MAVS) in the interaction with TRAF2, 3, 5, and 6 to activate both the NF-κB and IRF3 pathways. This study revealed that CSFV infection led to the upregulation of TRAF1 mRNA and protein levels; moreover, TRAF1 overexpression inhibited CSFV replication, while TRAF1 knockdown promoted replication, highlighting its importance in the host response to CSFV infection. Additionally, the expression of RIG-I, MAVS, TRAF1, IRF1, and ISG15 were detected in PK-15 cells infected with CSFV, revealing that TRAF1 plays a role in regulating IRF1 and ISG15 within the RIG-I pathway. Furthermore, Co-IP, GST pull-down, and IFA analyses demonstrated that TRAF1 interacted with MAVS and co-localized in the cytoplasm during CSFV infection. Ultimately, TRAF1 acted as a novel member of the TRAF family, bound to MAVS as a linker molecule, and functioned as a mediator downstream of MAVS in the RIG-I/MAVS pathway against CSFV replication.

## 1. Introduction

Classical swine fever virus (CSFV) causes a highly contagious disease of domestic pigs and wild boars. Persistent CSFV infection has been a significant detriment to the pig industry [1]. CSFV is a member of the Flaviviridae family and the Pestivirus genus and contains a single large open reading frame (ORF) with two untranslated regions (UTRs). The cleavage of the viral ORF by cellular and viral proteases results in the production of four structural proteins and eight non-structural proteins [2].

The tumor necrosis factor receptor (TNF-R)-associated factor (TRAF) family of scaffolding proteins, initially identified as signaling adaptors that directly interact with the cytoplasmic domains of TNF-R superfamily receptors, consists of seven members [3]. With the exception of TRAF7, all TRAF family members possess zinc finger, TRAF-N, and TRAF-C domains. TRAF1 is distinct from other TRAFs in its absence of the N-terminal RING finger domain [4,5]. TRAF proteins exhibit varying expression profiles, with TRAF2, 3, and 6 being widely expressed in most cell and tissue types [6,7,8]. In contrast, TRAF1 expression is restricted to specific tissues such as the tonsils, spleen, lungs, and testes and is tightly regulated. The porcine TRAF1 gene exhibits three highly sequentially similar transcribed variants, with minor differences in the N-terminal sequences [9]. As an essential member of the TRAF family, TRAF1 has significant expression under stimulation by TNF, interleukin 1, lipopolysaccharide, and lymphocyte receptors [10].

Innate immune pattern recognition receptors (PRRS) include retinoic acid-inducible gene I (RIG-I)-like receptors (RLRs) [11]. RIG-I, initially identified as a dsRNA binding protein involved in cell signaling and interferon (IFN) expression, has since been shown to recognize single (Flaviviridae viruses)- and double-stranded RNA viruses (Rotavirus, Dengue virus, West Nile virus) [12]. Upon recognition of viral RNA, RIG-I interacts with the CARD region of MAVS, leading to the formation of prion-like aggregates of other MAVSs on the outer mitochondrial membrane, which initiates signaling pathways [13].

MAVS consists of three domains: the N-terminal caspase recruitment domain (CARD), proline-rich domain (PRR), and C-terminal transmembrane domain (TM). It serves as a critical adaptor protein within the signaling pathway of RLRs and significantly contributes to the host antivirus immune response [14]. The CARD domain of MAVS interacts with the CARD domains of RIG-I and MDA5, while its PRR domain binds to TRAF2, 3, 5, and 6 members of the TRAF family, thereby facilitating subsequent signal transduction [15]. However, the function of TRAF1 within the RIG-I signaling pathway remains inadequately investigated. Increasing evidence has suggested that TRAF1 plays a crucial role in intracellular signal transduction [16]. TRAF1 is involved in the regulation of NF-κB substitution pathways in lymphocytes, which can recruit cIAPs to degrade TRAF3, leading to the activation of alternative pathways to NF-κB [17]. Accordingly, further exploration of the relationship between TRAF1 and the RIG-I signaling pathway or the potential interaction between TRAF1 and MAVS may offer a novel theoretical framework for managing CSFV infections.

A previous study by our group revealed that the activation of the RIG-I/MAVS pathway during CSFV infection resulted in the upregulation of downstream factors, IRF1 and ISG15. IRF1 translocated to the nucleus and bound to the –487 to –325 nucleotide region with the 5′ flanking region of ISG15, suggesting that ISG15 serves as a crucial downstream effector factor for IRF1 in exerting antiviral activity [18]. This study further demonstrated that CSFV infection induces TRAF1 upregulation, which plays an antiviral role by interacting with MAVS in the RIG-I pathway to enhance the expression of downstream signaling proteins IRF1 and ISG15, ultimately inhibiting CSFV replication.

## 2. Materials and Methods

### 2.1. Cells, Viruses, and Construction of Recombinant Plasmids

Porcine kidney (PK-15) and human embryonic kidney (HEK 293T) cells were maintained in Dulbecco’s modified Eagle’s medium (DMEM) supplemented with 10% fetal bovine serum and incubated at 37 °C with 5% CO_2_. A highly virulent CSFV Shimen strain was stored at –80 °C to be utilized in this study. The gene fragments encoding porcine TRAF1 and MAVS genes were amplified by reverse transcription–polymerase chain reaction (RT-PCR). The recombinant eukaryotic expression vectors pcDNA3.0-TRAF1^-Flag^ expressing the TRAF1 protein and pcDNA3.0-MAVS^-Myc^ expressing the MAVS protein were constructed in our laboratory using mammalian cell expression vectors with the CMV promotor. The recombinant prokaryotic expression vector PEGX 4T-1-MAVS^-GST^ expressing the MAVS protein was constructed in our laboratory using the expression vector of Escherichia coli carrying the Tac promoter and the GST label.

### 2.2. Antibodies and Reagents

Specific polyclonal antibodies were used in the Western blotting (WB) experiments. Anti-IRF1 (11335-1-AP), anti-RIG-I (20566-1-AP), anti-Myc tag (16286-1-AP), and anti-Flag tag (20543-1-AP) antibodies were purchased from Proteintech™ Co., Ltd. (Wuhan, China). Anti-CSFV E2 antibody (9011) was purchased from JBT™ Co., Ltd. (Seoul, Korea), and an anti-β-actin (CW0096A) antibody was purchased from CWBIO™ Co., Ltd. (Beijing, China). Horse anti-mouse IgG antibody (whole molecule) labeled with alkaline phosphatase (ZB-2310) and goat anti-rabbit IgG antibody (whole molecule) labeled with alkaline phosphatase (ZB-2308) were purchased from ZSGB™ Co., Ltd. (Beijing, China). Anti-ISG15 (Mouse) [19], anti-TRAF1 (Mouse) (CN202211672159.9), and anti-MAVS (Mouse) [20] antibodies were prepared in our laboratory. A BCIP/NBT Alkaline Phosphatase Color Development Kit (C3206-1), Glutathione (GSH) beads (P2258), and Protein A/G agarose beads (P2019) were purchased from Beyotime Co., Ltd. (Beijing, China). Lipofectamine 2000 reagent (2220839) was purchased from Invitrogen Co., Ltd. (Waltham, MA, USA).

### 2.3. Small Interfering RNA (siRNA) Knockdown

PK-15 cells were seeded into a 12-well plate and transfected with control siRNA or TRAF1-specific siRNA at a final concentration of 100 nM Lipofectamine 2000. After 24 h of siRNA transfection, the cells were infected with CSFV (Shimen strain) at a multiplicity of infection (MOI) of 1. The cell supernatants, cell extracts, and total RNA lysates were harvested at 24, 36, and 48 h post-infection (hpi) and analyzed by virus titration, WB, and RT-qPCR. The siRNA sequences are presented in Table 1.

### 2.4. Transfection/Infection Assays

The PK-15 cells were initially seeded into the 12-well plate, transfected with either the pcDNA3.0 plasmid or the recombinant eukaryotic expression plasmid pcDNA3.0-TRAF1^-Flag^ encoding TRAF1 (1.0 µg) for 6 h, and subsequently infected with CSFV (Shimen strain) at a MOI of 1. The supernatants and cell extracts were harvested at 24, 36, and 48 hpi. The released CSFV virions were quantified in duplicate using a fluorescent focus unit (FFU) assay [21] on the PK-15 cells (the PK-15 cells were infected by 10-fold serial dilution of CSFV Shimen strain, then detect the fluorescent focus by anti-CSFV E2 polyclonal antibody). Cellular and viral proteins were detected by WB analysis using specific antibodies. Total RNA from the infected cells was extracted using the standard TRIzol (Thermo Fisher Scientific, Cambridge, MA, USA) RNA extraction protocol for quantitative RT-PCR.

### 2.5. Immunoprecipitation Assay

HEK 293T cells were initially seeded in a 6-well plate and then transfected with the recombinant eukaryotic expression vectors pcDNA3.0-TRAF1^-Flag^ and pcDNA3.0-MAVS^-Myc^ using Lipofectamine 2000 reagent. At 36 h post-transfection (hpt), the cells were harvested and lysed, and the cell extracts were subjected to WB and co-immunoprecipitation analyses. Briefly, the cell extracts were incubated with either mouse IgG or anti-Flag (1:100 dilution)/anti-Myc (1:200 dilution) antibody overnight at 4 °C with continuous rotation. Protein A/G agarose beads were then added to the mixtures and incubated for 6 h with continuous rotation. After incubation, the beads were collected via centrifugation and washed five times. The input cell extracts and immunoprecipitates were then detected by WB analysis with mouse anti-TRAF1 (1:1000 dilution), mouse anti-MAVS (1:1000 dilution) antisera, or mouse anti-β-actin (1:2000 dilution) polyclonal antibodies.

### 2.6. Indirect Immunofluorescence Assay (IFA)

The cells were seeded onto glass coverslips in 35 mm cell culture dishes and cultured overnight. HEK 293T cells were then co-transfected with pcDNA3.0-TRAF1^-Flag^ and pcDNA3.0-MAVS^-Myc^. After 24 h, the cells were washed three times with cold phosphate buffer solution (PBS) and fixed with 4% paraformaldehyde for 20 min at room temperature. Subsequently, the cells were incubated with 4′,6-diamidino-2-phenylindole (DAPI) at 37 °C for 10 min and washed with cold PBS. Finally, images were captured by Nikon eclipse Ti fluorescence microscope (Japan).

### 2.7. WB Analysis

SDS-PAGE was performed using a 12% acrylamide gel in the Mini-protein Tetra system (Bio-Rad, Hercules, CA, USA) through a separation technique for protein samples, followed by transfer to a 0.45 μm polyvinylidene fluoride (PVDF) membrane. The non-specific proteins on the PVDF membrane were blocked overnight at 4 °C with 5% skim milk in Tris-buffered saline (TBS) containing 0.05% Tween-20 (TBS-T). The membranes were washed thrice with 1×TBS-T and incubated with primary antibodies at 37 °C for 1 h. After three more TBS-T washes, the membrane was incubated with an alkaline phosphatase-labeled secondary antibody for 1 h. Specific protein bands were visualized using the BCIP/NBT Alkaline Phosphatase Color Development Kit. The gray value of the target protein band from WB analysis corresponded to β-actin and was analyzed by comparing it to β-actin using NIH Image-J software, version 2.0.

### 2.8. Real-Time Quantitative Polymerase Chain Reaction (RT-qPCR)

The relative mRNA expressions of CSFV, TRAF1, TRAF2, TRAF3, TRAF4, TRAF5, TRAF6, RIG-I, MAVS, IRF1, and ISG15 were tested by RT-qPCR using specific primers and SYBR Green (Table 1). To measure target gene expression, total cellular RNA was isolated using the RNAsimple Total RNA Kit (Tiangen Biotech Co., Ltd., Beijing, China). First-strand complementary DNA (cDNA) was synthesized using the HiScript III RT SuperMix for qPCR (+gDNA wiper) kit (Vazyme Biotech Co., Ltd., Nanjing, China) according to the manufacturer’s instructions. A LightCycler 96 PCR detection system (Roche Diagnostic Co., Ltd., Shanghai, China) was used for the quantitative assessment of CSFV, TRAF1, TRAF2, TRAF3, TRAF4, TRAF5, TRAF6, RIG-I, MAVS, IRF1, and ISG15 mRNA under standard cycling conditions. GAPDH expression served as the reference gene for all reactions. Relative fold changes were calculated using the 2^−ΔΔCt^ method.

### 2.9. Statistical Analysis

All experiments were repeated at least three times. Data were analyzed by GraphPad Prism software, version 8.0 (GraphPad Software, La Jolla, CA, USA), and OriginPro software, version 9.1 (OriginLab, Northampton, MA, USA). The statistical differences between groups were analyzed using the Student’s t-test or one-way analysis of variance. Correlations between quantitative variables were assessed using Pearson’s correlation coefficient. A probability (*p*) value of less than 0.05 was considered to be statistically significant. Correlation coefficient |R| ≥ 0.9—very strong; 0.7 ≤ |R| < 0.9—strong; 0.5 ≤ |R| < 0.7—moderate; 0.3 ≤ |R| < 0.5—weak; |R| < 0.3—very weak.

## 3. Results

### 3.1. CSFV Infection Significantly Upregulated TRAF1 in the TRAF Family

In our previous research, CSFV was shown to activate the RIG-I/MAVS pathway and upregulate the downstream factors IRF1 and ISG15 [18]. All members except for TRAF7 contain a TRAF domain that mediates interactions between various types of intracellular proteins [22]. We speculated that one member of the TRAF family with a TRAF domain may serve as a critical adaptor protein involved in the RIG-I/MAVS pathway.

**Figure 1:**
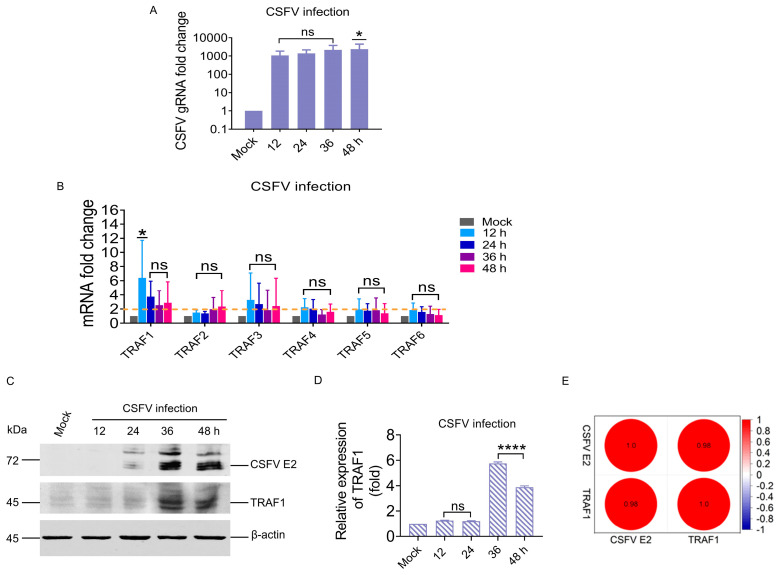
CSFV infection significantly upregulated TRAF1 in TRAF family. (**A**) CSFV replication in PK-15 cells. RT-qPCR analyzed CSFV gRNA levels at 12, 24, 36, and 48 hpi in CSFV-infected (MOI = 1) PK-15 cells. (**B**) The mRNA levels of TRAF1, TRAF2, TRAF3, TRAF4, TRAF5, and TRAF6 proteins were detected in CSFV-infected (MOI = 1) PK-15 cells by RT-qPCR analysis at 12, 24, 36, and 48 hpi. (**C**) The protein expression levels of CSFV E2 and TRAF1 in CSFV-infected (MOI = 1) PK-15 cells were assessed by WB analysis at 12, 24, 36, and 48 hpi. (**D**) The gray values of TRAF1 band from WB were measured by comparing them with β-actin using ImageJ software version 1.8.0. (**E**) Pearson analysis of the CSFV E2 and TRAF1 protein bands. All statistical analysis results were derived from comparisons between the experimental group and the Mock group (* *p* < 0.05, **** *p* < 0.0001).

To understand the effect of CSFV infection on host protein TRAFs, members of the TRAF family without TRAF7 were assessed in PK-15 cells infected with CSFV. This study examined the mRNA levels of TRAF1 to TRAF6 protein at various time points (12, 24, 36, and 48 h) following CSFV infection in PK-15 cells using RT-qPCR. The results indicated a substantial increase in CSFV replication within PK-15 cells, with fold changes of 1100.7, 1422.2, 2202.6, and 2432.6 (*p <* 0.05) at 12, 24, 36, and 48 hpi, respectively (Figure 1A). The mRNA levels of TRAF1 and TRAF3 proteins were more than two-fold higher following CSFV infection, with TRAF1 showing the most prominent increase of 6.3- (*p <* 0.05), 3.7-, 2.4-, and 2.8-fold at 12, 24, 36, and 48 hpi, respectively. Conversely, no significant changes were observed in the mRNA levels of TRAF2, TRAF4, TRAF5, and TRAF6 during CSFV infection (Figure 1B). Simultaneously, the correlation between CSFV and TRAF1 was measured at the protein level using WB analysis. TRAF1 protein levels were detected in PK-15 cells infected with CSFV; however, due to the presence of three highly similar transcript variants of the TRAF1 gene, multiple bands with a molecular weight of approximately 45 kDa were observed, making it challenging to distinguish individual protein levels. Quantification was conducted for both TRAF1 and its transcript variants. The results revealed that the expression of the TRAF1 protein was upregulated 5.7- (*p <* 0.0001) and 3.8-fold (*p <* 0.0001) at 36 and 48 hpi, respectively (Figure 1C,D). In addition, Pearson analysis of the CSFV E2 and TRAF1 protein bands showed that there was a significant positive correlation between CSFV E2 and TRAF1 protein (Figure 1E). These results indicated that CSFV infection can activate TRAF1 in the TRAF family, and the expression of TRAF1 is positively correlated with CSFV infection.

### 3.2. TRAF1 Expression Positively Correlated with the RIG-I/MAVS Pathway during CSFV Infection

The above experiment showed that CSFV infection significantly induced TRAF1 expression.

**Figure 2:**
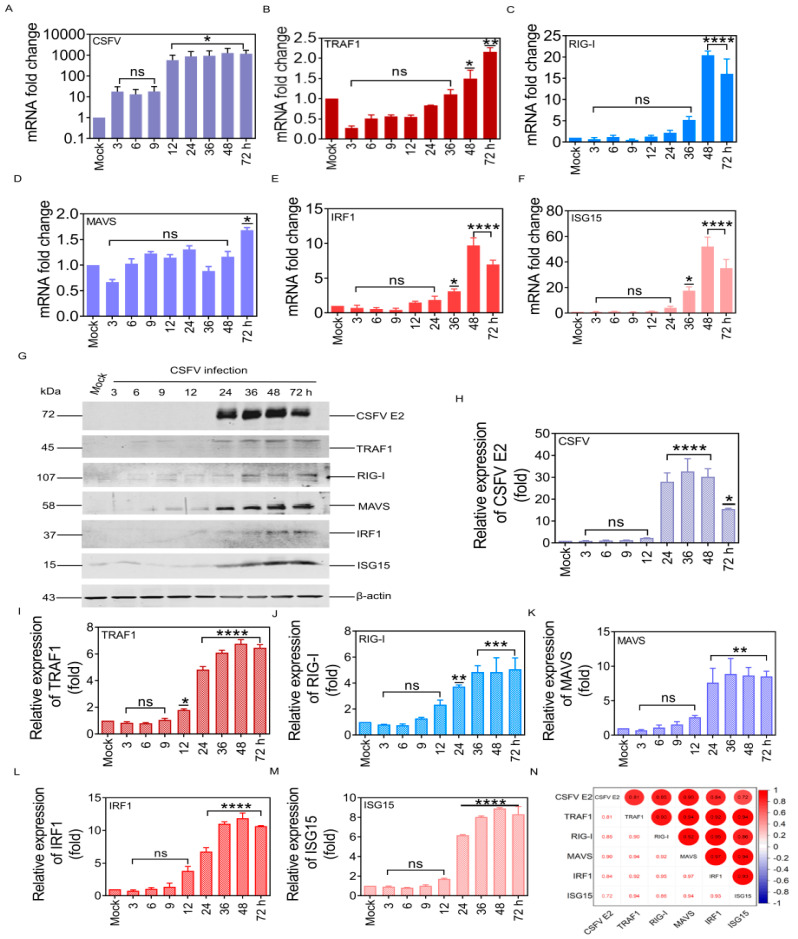
TRAF1 expression positively correlated with the RIG-I/MAVS pathway during CSFV infection. (**A**–**F**) The CSFV gRNA and the mRNA levels of TRAF1, RIG-I, MAVS, IRF1, and ISG15 proteins were detected in PK-15 cells infected with the CSFV Shimen strain (MOI = 1) at 3, 6, 9, 12, 24, 36, 48, and 72 hpi by RT-qPCR analysis. (**G**) The protein levels of CSFV E2, TRAF1, RIG-I, MAVS, IRF1, and ISG15 were detected in PK-15 cells infected with the CSFV Shimen strain (MOI = 1) at 3, 6, 9, 12, 24, 36, 48, and 72 hpi by WB analysis. (**H**–**M**). (**N**) Pearson analysis of the CSFV E2, TRAF1, RIG-I, MAVS, IRF1, and ISG15 protein bands. The gray value of each target band from WB was measured by comparing them to β-actin using ImageJ software version 1.8.0. All statistical analysis results were derived from comparisons between the experimental group and the Mock group (* *p* < 0.05, ** *p* < 0.01, *** *p* < 0.001, **** *p* < 0.0001).

To further elucidate the significance of TRAF1 in the RIG-I/MAVS pathway during CSFV infection, the mRNA and protein levels of TRAF1, RIG-I, MAVS, IRF1, and ISG15 in PK-15 cells infected with 1.0 MOI CSFV were assessed by RT-qPCR and WB analyses. A substantial increase in CSFV genomic RNA (gRNA) expression was observed, resulting in upregulation of 866.3 (*p <* 0.05), 933.3 (*p <* 0.05), 1259.9 (*p <* 0.05), and 1165.9-fold (*p <* 0.05) at 24, 36, 48, and 72 hpi, respectively (Figure 2A). Furthermore, higher mRNA levels of TRAF1, RIG-I, MAVS, IRF1, and ISG15 proteins were detected in CSFV-infected PK-15 cells at 24, 36, 48, and 72 hpi, respectively, compared with the mock group (Figure 2B–F). Specifically, TRAF1 mRNA levels were progressively upregulated at 36 and 48 hpi and upregulated to 2.1-fold (*p <* 0.01) at 72 hpi (Figure 2B). Additionally, RIG-I mRNA levels were upregulated to 2.1-fold at 24 hpi, 5.1-fold at 36 hpi, 20.3-fold (*p <* 0.0001) at 48 hpi, and 16.0-fold (*p <* 0.0001) at 72 hpi (Figure 2C). MAVS mRNA levels were progressively upregulated at 48 and 72 hpi (Figure 2D), while IRF1 mRNA levels were upregulated by 3.0-fold (*p <* 0.05) at 36 hpi, 9.6-fold (*p <* 0.0001) at 48 hpi, and 6.9-fold (*p <* 0.0001) at 72 hpi (Figure 2E). ISG15 mRNA levels were upregulated by 4.0-fold at 24 hpi, 17.4-fold (*p <* 0.05) at 36 hpi, 52.0-fold (*p <* 0.0001) at 48 hpi, and 35.0-fold (*p <* 0.0001) at 72 hpi (Figure 2F).

Simultaneously, the protein expression of CSFV E2, TRAF1, RIG-I, MAVS, IRF1, and ISG15 were assessed in PK-15 cells infected with CSFV (MOI = 1) by WB analysis. The results revealed a gradual increase in TRAF1, RIG-I, MAVS, IRF1, and ISG15 protein levels following CSFV infection. Additionally, the presence of TRAF1, RIG-I, MAVS, IRF1, and ISG15 protein bands was only detected when the CSFV E2 protein band was present (Figure 2G–M). The detailed fold changes of CSFV E2, TRAF1, RIG-I, MAVS, IRF1, and ISG15 proteins at different time points after CSFV infection are shown in Table 2. Pearson correlation coefficient analysis was used to examine the correlations between TRAF1, RIG-I, MAVS, IRFI, ISG15, and CSFV E2 protein expression. The findings indicated a positive correlation of CSFE E2 with TRAF1, RIG-I, MAVS, IRF1, and ISG15. Furthermore, TRAF1 protein exhibited a positive correlation with RIG-I, MAVS, IRF1, and ISG15 protein (Figure 2N).

In summary, these results demonstrated that infection with a virulent CSFV Shimen strain can induce the expressions of TRAF1, RIG-I, MAVS, IRF1, and ISG15. Furthermore, there was a positive correlation between TRAF1 expression and the RIG-I/MAVS pathways.

### 3.3. Interaction of TRAF1 with MAVS

**Figure 3:**
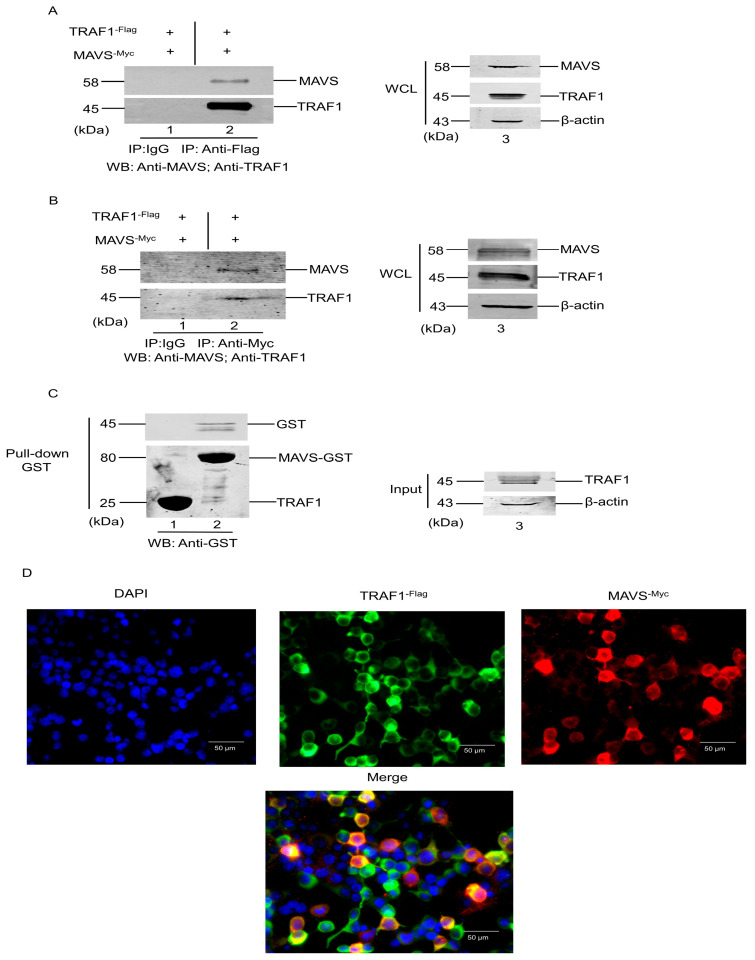
TRAF1 interacts with MAVS. (**A**) The expression of TRAF1 and MAVS proteins was analyzed in HEK 293T cells through co-transfection of 1 µg of pcDNA3.0-TRAF1^-Flag^ and pCMV-MAVS^-Myc^ vectors, followed by WB analysis at 36 hpi. A portion of the cell extract was utilized for assessing TRAF1 and MAVS protein expression in total cells (lane 3), while the remainder was subjected to Co-IP analysis using anti-TRAF1 antibody (lane 2) or normal mouse IgG (lane 1). (**B**) Most experimental procedures were the same as above, except that the remainder was subjected to Co-IP analysis using anti-MAVS antibody (lane 2) or normal mouse IgG (lane 1). (**C**) Purified GST and GST-MAVS fusion proteins from the recombinant prokaryotic expression systems were incubated with pcDNA3.0-TRAF1^-Flag^ transfected HEK-293T cell lysate, TRAF1, MAVS, and GST control in Pull-down samples were detected by WB analysis using specific antibodies. (**D**) The interaction between TRAF1 and MAVS was observed in the cytoplasm. The co-transfection of 1 µg of pcDNA3.0-TRAF1^-Flag^ and pCMV-MAVS^-Myc^ vectors into PK-15 cells was analyzed using indirect immunofluorescence at 36 hpi.

As described in Section 3.2, TRAF1 expression was significantly increased and played an important role in the RIG-I/MAVS pathway in response to CSFV infection. The interactions between TRAF2, TRAF3, TRAF5, and TRAF6 with MAVS have also been confirmed [14]. Herein, a hypothesis has been proposed, suggesting that TRAF1 interacts with MAVS to contribute to the RIG-I/MAVS pathway. This potential interaction was investigated using a co-immunoprecipitation (Co-IP) assay, GST pull-down, and fluorescence microscopy. Briefly, HEK 293T cells were transfected with plasmids encoding Flag-tagged TRAF1 and Myc-tagged MAVS, followed by immunoprecipitation using anti-Flag antibody or normal mouse IgG and subsequent WB analysis. As demonstrated in Figure 3A, MAVS protein was detected in the TRAF1 protein immunoprecipitates but not in the corresponding IgG control precipitates (Figure 3A, compare lanes 1 and 2), suggesting an interaction between exogenous MAVS and TRAF1 in HEK 293T cells.

Additional experiments were conducted to further confirm this interaction. Briefly, HEK 293T cells were transfected with plasmids encoding Flag-tagged TRAF1 and Myc-tagged MAVS, followed by immunoprecipitation using anti-Myc antibody or normal mouse IgG and subsequently analyzed by WB. As presented in Figure 3B, TRAF1 protein was detected in the MAVS protein immunoprecipitates but not in the corresponding IgG control precipitates (Figure 3B, compare lanes 1 and 2). Taken together, these results demonstrated that TRAF1 is a specific host protein interactor for MAVS protein.

Furthermore, the GST-tagged MAVS protein was expressed and purified using the recombinant prokaryotic expression system PEGX 4T-1-MAVS-GST to create a bait by conjugating GST-MAVS with GSH beads. This bait was then incubated with whole cell lysates from pcDNA3.0-TRAF1-Flag-transfected HEK-293 cells to isolate MAVS-interacting TRAF1 proteins (Figure 3C, lane 3). TRAF1 was identified in the GST-MAVS samples but not in the GST protein control, indicating that TRAF1 can directly interact with MAVS in vitro (Figure 3C, compare lanes 1 and 2).

The previous findings indicated that TRAF1 interacted with MAVS, prompting further investigation into the co-localization of TRAF1 with MAVS in cells. Fluorescence microscopy was utilized to examine HEK 293T cells co-transfected with TRAF1-Flag and MAVS-Myc proteins. Microscopy analysis revealed the co-localization of TRAF1 with MAVS within the cytoplasm; despite the diffused distribution of TRAF1 in this cellular compartment, it co-localized with MAVS (Figure 3D). These findings, in conjunction with previous Co-IP and GST pull-down experiments, provide evidence for an interaction between TRAF1 and MAVS.

### 3.4. TRAF1 Downregulation by siRNA-Enhanced CSFV Replication

The above results have demonstrated that CSFV infection upregulated TRAF1 expression, increasing its interaction with MAVS in the RIG-I/MAVS pathway. To investigate the impact of TRAF1 silencing on CSFV replication, TRAF1 siRNA and a control siRNA were transfected into CSFV-infected PK-15 cells. The mRNA and protein levels of TRAF1, CSFV E2, RIG-I, MAVS, IRF1, and ISG15 were assessed using RT-qPCR and WB analysis, whereas virus titration was determined through IFA.

**Figure 4:**
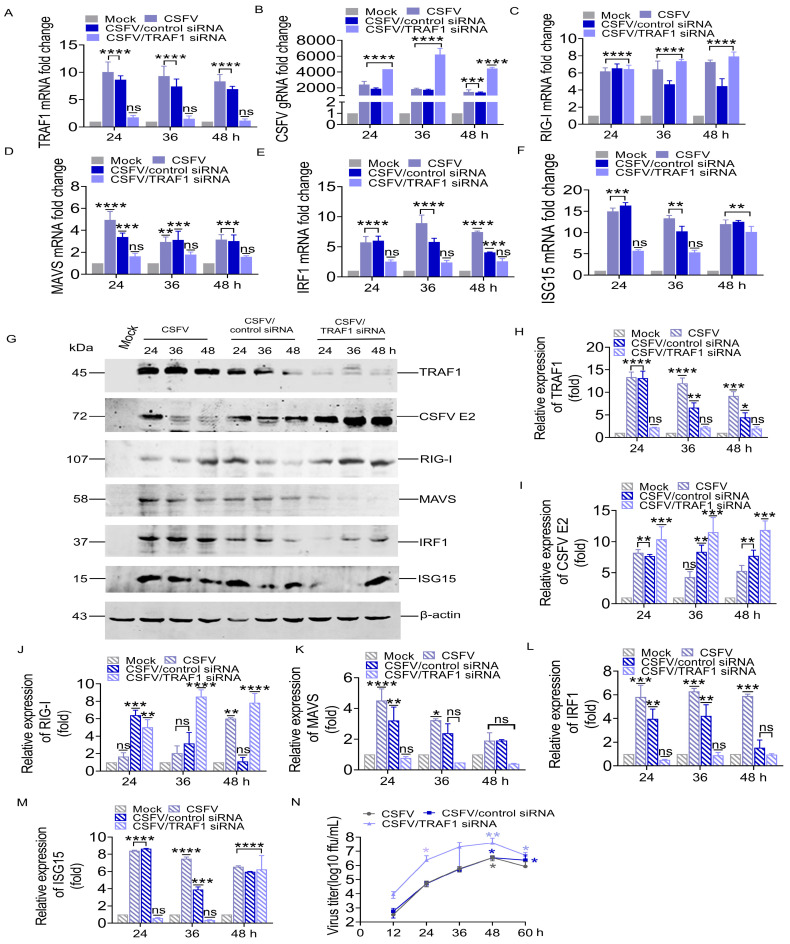
TRAF1 downregulation by siRNA-enhanced CSFV replication. (**A**–**F**) The CSFV gRNA and the mRNA levels of TRAF1, RIG-I, MAVS, IRF1, and ISG15 proteins were detected in different kinds of PK-15 cells, such as infection of CSFV Shimen strain at an MOI of 1.0 as a positive control, temporary transfection of 100 nM TRAF1 siRNA 6 h then infection of CSFV Shimen strain, temporary transfection of 100 nM control siRNA 6 h then infection of CSFV Shimen strain as a negative control by RT-qPCR analysis at 24, 36, and 48 hpi. (**G**) The experimental treatment was consistent with the (**A**) and WB analysis at 24, 36, and 48 hpi. (**H**–**M**) The gray value of each target band from WB was measured by comparing them to β-actin using ImageJ software version 1.8.0. (**N**) The experimental treatment was consistent with (**A**). Culture supernatant from PK-15 cells subjected to different treatments was collected, and the viral titer was tested by IFA at 12, 24, 36, 48, and 60 hpi. All statistical analysis results were derived from comparisons between the experimental group and the Mock group (* *p* < 0.05, ** *p* < 0.01, *** *p* < 0.001, **** *p* < 0.0001).

In PK-15 cells transfected with 100 nM control siRNA and infected with CSFV (MOI = 1), the expression of TRAF1, RIG-I, MAVS, IRF1, and ISG15 increased with viral replication (Figure 4A–F,H–M). However, PK-15 cells transfected with 100 nM TRAF1-specific siRNA and infected with CSFV (MOI = 1) exhibited significant suppression of endogenous TRAF1 mRNA level. Specifically, the TRAF1 mRNA expression decreased by 5.0, 4.9, and 6.1-fold compared to PK-15 cells transfected with control siRNA and infected with CSFV (control siRNA group) at 24, 36, and 48 h, respectively (Figure 4A), whereas TRAF1 protein levels could not be detected in TRAF1-specific siRNA-treated PK-15 cells (Figure 4G,H). A significant increase in the levels of CSFV gRNA and CSFV E2 protein was observed to be 2.3, 3.5, and 3.2-fold, and 1.3, 1.3, and 1.5-fold compared to the control siRNA group at 24, 36, and 48 h, respectively (Figure 4B,G,I).

Additionally, it was observed that the levels of RIG-I mRNA and protein, which are located upstream of TRAF1, remained unaffected by TRAF1 knockdown. It exhibited a significant increase with CSFV infection, compared to the control siRNA group, RIG-I mRNA level upregulated by 1.5 and 1.7-fold (Figure 4C), whereas the RIG-I protein level upregulated by 2.7 and 7.7-fold (Figure 4G,J) at 36 and 48 h, respectively. However, the mRNA and protein levels of MAVS, IRF1, and ISG15 decreased when the downregulation of TRAF1 expression by TRAF1-specific siRNA treatment. In detail, compared to the control siRNA group, the mRNA level of MAVS was decreased by 2.3, 1.8, and 1.9-fold (Figure 4D), while the MAVS protein level was decreased by 4.4, 5.75, and 4.5-fold at 24, 36, and 48 h, respectively (Figure 4G,K). The IRF1 mRNA level decreased by 2.4, 2.4, and 1.6-fold (Figure 4E), while the IRF1 protein level decreased by 7.8, 4.5, and 1.5 fold at 24, 36, and 48 h, respectively (Figure 4G,L). The ISG15 mRNA level decreased by 4.2 and 1.9-fold (Figure 4F), while the ISG15 protein level decreased by 17.2 and 13.0-fold at 24 and 36 h, respectively (Figure 4G,M).

Notably, the results of IFA showed a significant increase in virus titers in the supernatants of the cells receiving TRAF1-specific siRNAs compared to those receiving control siRNA. Interestingly, while the virion yield showed a step-by-step increase along with the infection time from the control cells, the knockdown of the endogenous TRAF1 substantially enhanced the virus infection and release (Figure 4N).

In summary, these siRNA silencing results confirm that TRAF1 negatively regulates virus infection and release by interacting with the MAVS protein and is involved in the RIG-I pathway.

### 3.5. TRAF1 Overexpression Inhibits CSFV Replication by the Positive Regulation of IRF1 and ISG15

These results indicate that TRAF1 silencing by specific siRNA significantly promoted the infection and release of CSFV. To further verify the effect of TRAF1 on CSFV replication and explore the underlying mechanism, the pcDNA3.0-TRAF1^-Flag^ eukaryotic vector was transfected into CSFV-infected PK-15 cells. The mRNA and protein levels of TRAF1, CSFV E2, RIG-I, MAVS, IRF1, and ISG15 were assessed by RT-qPCR and WB analyses, while virus titration was determined through IFA.

**Figure 5:**
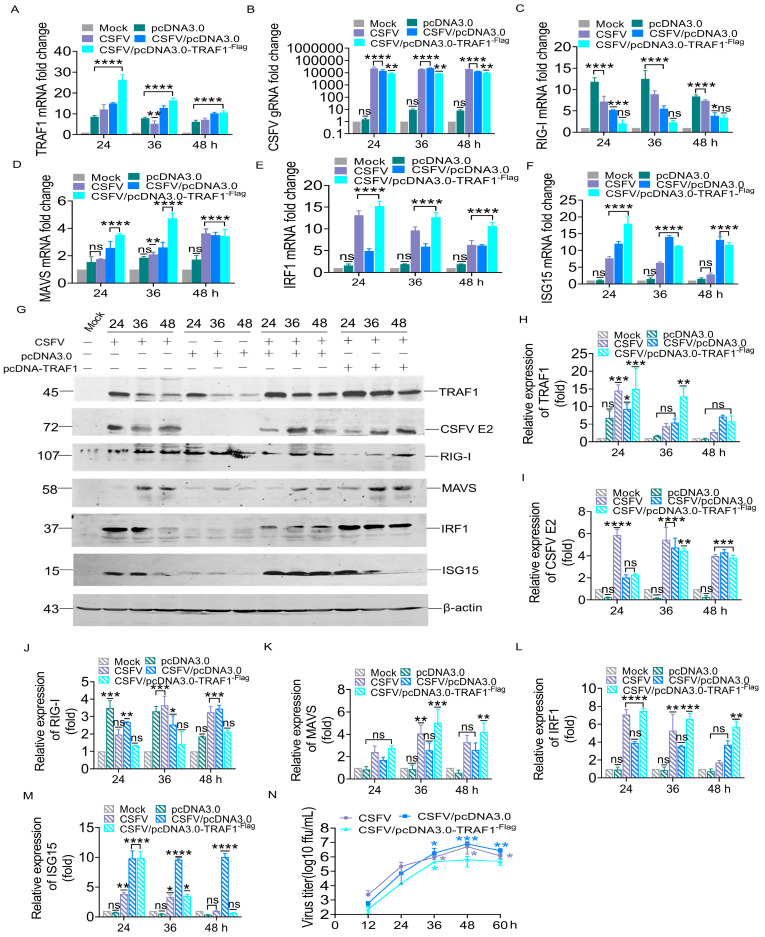
TRAF1 overexpression inhibited CSFV replication by positive regulation of IRF1 and ISG15. (**A**–**F**) The CSFV gRNA and mRNA levels of TRAF1, RIG-I, MAVS, IRF1, and ISG15 proteins were detected in different kinds of PK-15 cells, such as infection of CSFV Shimen strain at an MOI of 1.0 as a positive control, temporary transfection of 1.0 µg pcDNA3.0-TRAF1^-Flag^ vector 12 h then infection of CSFV Shimen strain, temporary transfection of 1.0 µg empty vector 12 h then infection of CSFV Shimen strain as negative control by RT-qPCR analysis at 24, 36, and 48 hpi. (**G**) The experimental treatment was consistent with (**A**) and the WB analysis at 24, 36, and 48 hpi. (**H**–**M**) The gray value of each target band from WB was measured by comparing them to β-actin using ImageJ software version 1.8.0. (**N**) The experimental treatment was consistent with (**A**). Culture supernatant from PK-15 cells subjected to different treatments was collected, and the viral titer was tested by IFA at 12, 24, 36, 48, and 60 hpi. All statistical analysis results were derived from comparisons between the experimental group and the Mock group (* *p* < 0.05, ** *p* < 0.01, *** *p* < 0.001, **** *p* < 0.0001).

In PK-15 cells transfected with 1.0 μg empty vector and infected with CSFV (MOI = 1), the expression of TRAF1, RIG-I, MAVS, IRF1, and ISG15 increased with viral replication (Figure 5A–F,H–M). However, PK-15 cells transfected with 1.0 μg pcDNA3.0-TRAF1^-Flag^ vector and infected with CSFV (MOI = 1) showed that the exogenous expression of TRAF1 protein was significantly enhanced. Specifically, the TRAF1 mRNA level increased by 1.7-fold at 24 h, 1.2-fold at 36 h, and 1.0-fold at 48 h compared to PK-15 cells transfected with 1.0 μg empty vector and infected with CSFV (empty vector group), whereas TRAF1 protein levels increased by 1.6-fold at 24 h and 2.3-fold at 36 h (Figure 5A,G–H). In contrast, a significant decrease in the levels of CSFV gRNA and CSFV E2 protein was observed to be 1.6, 2.8, and 1.3-fold at 24, 36, and 48 h, and 1.0, and 1.1-fold at 36 and 48 h compared to the empty vector group, respectively (Figure 5B,G,I).

Additionally, it was observed that the levels of RIG-I mRNA and protein, which are located upstream of TRAF1, remained unaffected by TRAF1 overexpression. Compared to the empty vector group, the RIG-I mRNA level decreased by 2.5, 2.4 and 1.1-fold (Figure 5C), whereas the RIG-I protein level decreased by 2.0, 1.7, and 1.7-fold (Figure 5G,J) at 24, 36, and 48 h, respectively. In contrast, the mRNA and protein levels of MAVS, IRF1, and ISG15 were observed to increase in response to TRAF1 expression upregulation induced by transfection with the pcDNA3.0-TRAF1^-Flag^ eukaryotic vector in CSFV-infected PK-15 cells. In detail, compared to the empty vector group, the mRNA level of MAVS increased by 1.4, 1.8, and 1.0 fold (Figure 5D), while MAVS protein levels were increased by 1.6, 2.0, and 1.6-fold at 24, 36, and 48 h, respectively (Figure 5G,K). The mRNA level of IRF1 increased by 3.1, 2.1, and 1.7-fold (Figure 5E), while the IRF1 protein level increased by 1.8, 1.9, and 1.5-fold at 24, 36, and 48 h, respectively (Figure 5G,L). The mRNA level of ISG15 increased by 1.4-fold at 24 h (Figure 5F).

Intriguingly, the results of IFA showed a significant reduction in the yield of progeny virions upon the addition of exogenous TRAF1 (Figure 5N), implying that the addition of exogenous TRAF1 inhibited CSFV infection, and this antivirus effect might be due to a part of the RIG-I/MAVS pathway fighting against CSFV infection.

## 4. Discussion

Innate immunity is the body’s first immune barrier of defense against foreign pathogens, with PRRS, such as Toll-like receptors (TLRs), RLRs, and NOD-like receptors (NLRs), playing a crucial role in detecting pathogen-associated molecular patterns (PAMPs) expressed by invading viruses [23]. Upon activation of RIG-I/MDA5, the interaction between their CARD domains and MAVS’s CARD domains triggers downstream signaling cascades involving TANK-binding kinase 1 (TBK1) and nuclear factor kappa-B kinase epsilon (IKKε), ultimately leading to the activation of interferon regulators IRF3 and IRF7 as well as NF-κB, thereby impeding viral replication and dissemination [11].

TRAF plays an important role in mediating the activation of IRF3 and IRF7 and the enucleation of NF-κB [24]. The members of the TRAF family, including TRAF2, TRAF3, TRAF5, and TRAF6, bind to the PRR domain of MAVS to facilitate the activation of the TBK1 complex, leading to the phosphorylation and homodimerization of IRF3 and IRF7 and their subsequent translocation to the nucleus and inducing the expression of type I interferons [15]. Moreover, TRAF2, TRAF5, and TRAF6 also interact with MAVS to promote the activation of the IKKε complex, which in turn, activates NF-κB to enhance the transcription of proinflammatory factors and participate in antiviral immunity.

Recently, more progress has been made in TRAF1 research. TRAF1 inhibits CD3-induced NF-κB activation and proliferation in T cells [25]. TRAF1 overexpression inhibits TRIF- and TLR3-mediated activation of NF-κB and expression of IFNβ, suggesting that TRAF1 inhibits TRIF-dependent signaling [26]. Phosphorylation of TRAF1 (at Ser 139 in mice and Ser 146 in humans) by PKN1 inhibits TNF-R2-dependent NF-κB and JNK signaling in HeLa cells and negatively impacts the recruitment of TBK1 to the TNFR family member 4-1BB signaling complex and the subsequent NF-κB activation in T cells [27]. Hyperproliferation of TRAF1^–/–^ T cells is due to constitutive activation of the NF-κB2 pathway [28]. TRAF1-deficient dendritic cells stimulated with CD154 exhibit impaired NF-κB activation and reduced survival, along with enhanced degradation of TRAF2 upon CD154 re-stimulation [29]. The findings of this study confirmed that CSFV infection causes TRAF1 upregulation but does not impact TRAF2, TRAF4, TRAF5, or TRAF6 levels (Figure 1). Furthermore, changes in TRAF1 mRNA and protein expression during CSFV infection were positively correlated with RIG-I, MAVS, IRF1, and ISG15 (Figure 2), suggesting that TRAF1 plays a vital role during CSFV infection by its involvement in the RIG-I pathway. Additionally, TRAF3 and TRAF6 participate in the innate immune response via the RIG-I pathway [30,31]. It was hypothesized that MAVS may recruit TRAF1 as a substitute factor in the RIG-I pathway to facilitate the activation of IRF1 and ISG15 expression.

With the exception of TRAF7, members of the TRAF family exhibit highly conserved C-terminal motifs known as TRAF domains, which facilitate interactions between TRAF members and specific intracellular proteins or signaling molecules [4]. Studies in the past decade have revealed that TRAF1 can bind to various intracellular proteins, including linker proteins (TRADD, TANK, and TRIP), NIK, RIP, RIP2, cIAP1, and cIAP2 [32]. MAVS is a crucial mitochondrial antiviral protein involved in host cell innate immunity and various viral immune signaling pathways [33]. To investigate the potential interaction between MAVS and TRAF1, Co-IP, GST pull-down, and laser confocal experiments were conducted. The results revealed that TRAF1 interacts with MAVS in the cytoplasm (Figure 3). The PRR domain of MAVS contains various TRAF binding sites, known as TRAF action units, and the PRR domain can recruit these proteins to form MAVS aggregates [34]. The interaction between TRAF1 and MAVS may occur through the PRR domain of MAVS; however, the precise mechanism of TRAF1 binding to MAVS and their interaction remains unclear.

To delve deeper into the effect of TRAF1 on viral replication and the relationship between TRAF1 and the RIG-I pathway, TRAF1 was both overexpressed and knocked down in PK-15 cells. This study confirmed that TRAF1 exhibits an antiviral function, as the overexpression of TRAF1 led to a significant decrease in CSFV transcription level, protein level, and progeny virus titers (Figure 5), while TRAF1 knockdown significantly increased these parameters (Figure 4). Recently, an increasing number of studies have highlighted the involvement of the TRAF family in viral infections. In a cross-sectional cohort, TRAF1 was highly expressed in CD8T cells from patients responding to HIV, and its protein level was higher in HIV-specific CD8T cells from patients who were able to control HIV in the absence of drug treatment [35]. TRAF1 expression is positively correlated with the expression levels of IL-7R, myeloid cell leukemia-1, and CD107a and is involved in supporting specific CD8^+^ T cell responses during hepatitis C virus infection [36]. Latent membrane protein 1 recruits TRAF proteins, including TRAF1, to mimic CD40 receptor signaling in Epstein–Barr virus (EBV)-infected B lymphocytes, leading to the activation of NF-κB, MAPK, IRF7, and STAT pathways [37]. FK506-binding protein 51 (FKBP51) interacts with TRAF6 to promote the expression of type I interferon and inhibit the infection of Newcastle disease virus [38]. HIV-1 Tat directly binds to TRAF6 to disrupt the host transcriptional pathway in order to enhance its own transcription [39]. Additionally, Goli protein 73 (GP73) promotes MAVS/TRAF6 degradation by interacting with the MAVS/TRAF6 complex and promotes HCV infection [40]. Additionally, the results demonstrated that TRAF1, acting downstream of RIG-I, plays an important role in modulating the expression of IRF1 and ISG15. The expression levels of MAVS, IRF1, and ISG15 were positively correlated with TRAF1 (Figure 4 and Figure 5). Overexpression of TRAF1 was recruited around MAVS to form aggregates, which promoted the upregulation of MAVS expression (Figure 5). When TRAF1 was knocked down, its binding to MAVS decreased; therefore, MAVS expression was also reduced (Figure 4). The signaling pathway complexity was evident, as CSFV induced the upregulation of ISG15 expression, while the antiviral protein ISG15 provided feedback regulating CSFV replication [18]. The interaction of MAVS with TRAF3/6 plays a crucial role in various viral infection mechanisms by activating the IRF3, IRF7, and NF-kB pathways. Our results focus on the activation of IRF1/ISG15 by the MAVS/TRAF1 complex. Understanding this mechanism will help in exploring host immune responses triggered by other viruses that also involve non-responsive IRF3, IRF7, and NF-kB pathways. Further investigation is required to elucidate the impact of the combined presence of TRAF1 and MAVS on the regulation of the signaling pathway.

## 5. Conclusions

**Figure 6:**
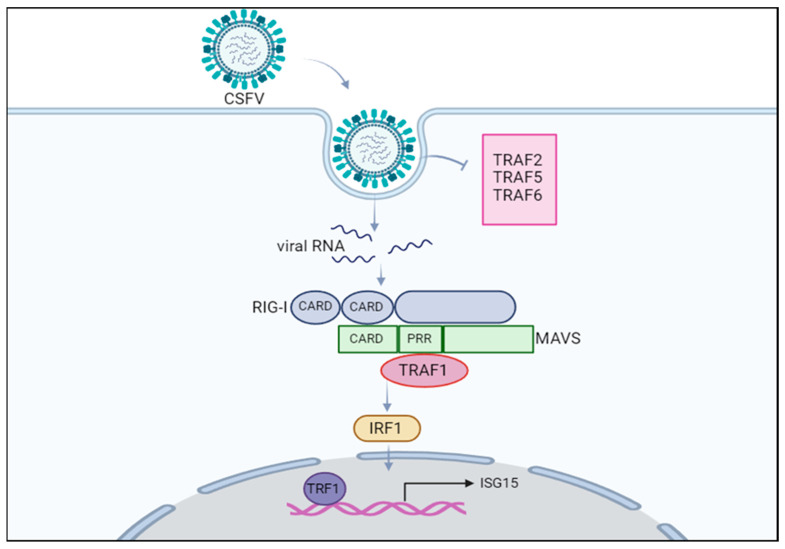
TRAF1 participated in the RIG-I/MAVS pathway against CSFV infection. RIG-I identifies short plus-stranded RNA of CSFV. After activation of RIG-I, its CARD region merges with the CARD region of MAVS to activate downstream signal molecules. TRAF1 binds to the PRR domain of MAVS to promote IRF1 activation and subsequent stimulation of ISG15 expression.

This study is the first report to reveal the role of TRAF1 interacting with MAVS of the RIG-I pathway to upregulate IRF1/ISG15 expression in the battle against CSFV infection (Figure 6).

## Figures and Tables

**Table 1:** Primers used in this study.

Primers	Sequence (5′-3′)	Purpose
CSFV-F	GCCATGCCCATAGTAGGACT	detection of CSFV gRNA
CSFV-R	GCTTCTGCTCACGTCGAACT	
RIG-I-F	CACATTTGCGGATATACAAC	detection of RIG-I mRNA
RIG-I-R	TCGGGCATGTTCATTGATAA	
MAVS-F	CTGGAGATTCTGCCTTACCTGT	detection of MAVS mRNA
MAVS-R	CAGATCCTCAGTGCCCCGAT	
TRAF1-F	AATGCAAAGGCGACGACACTCC	detection of TRAF1 mRNA
TRAF1-R	CCAACACCAGCAAAAGGGCATC	
TRAF2-F	CGCTCTTCTGCCCCGTCT	detection of TRAF2 mRNA
TRAF2-R	TAGAGCCCCGTCAGGTCCAC	
TRAF3-F	ACAGCGAGTCATAGACAGCC	detection of TRAF3 mRNA
TRAF3-R	TCCACGCTGCTCTTCATGCT	
TRAF4-F	GCAGCTTCAATGTGGTTCCCT	detection of TRAF4 mRNA
TRAF4-R	AAGTCACAGCCACAGAACTCG	
TRAF5-F	TCTTCAGCCAGCCCTTCTAC	detection of TRAF5 mRNA
TRAF5-R	TCCCCATTCAGGTACGCTCT	
TRAF6-F	CAAAGCCTGCATCATCAAGTC	detection of TRAF6 mRNA
TRAF6-R	ATTTGGACACTTCACCGTCAG	
IRF1-F	AGTCCAAGTCCAGCCGAGAT	detection of IRF1 mRNA
IRF1-R	TACTGATGGCACACGGTGAC	
ISG15-F	GCCTTCCAGCAGCGTCT	detection of ISG15 mRNA
ISG15-R	GCGTTGCTGCGACCCT	
GAPDH-F	TGGTGAAGGTCGGAGTGAAC	detection of GAPDH mRNA
GAPDH-R	GGAAGATGGTGATGGGATTTC	
TRAF1 siRNA-F	GCCUGCGGCUCUACCUGAATT	Knockdown of TRAF1
TRAF1 siRNA-R	UUCAGGUAGAGCCGCAGGCTT	

**Table 2:** Fold-changes of the levels of CSFV E2, TRAF1, RIG-I, MAVS, and ISG15 proteins in PK-15 cells following CSFV infection.

Protein Name	Fold Changes to Protein Levels of PK-15 Cells Infection with CSFV (MOI = 1)			
	24 hpi	36 hpi	48 hpi	72 hpi
CSFV E2	27.9	32.6	30.1	15.4
TRAF1	4.7	6.0	6.7	6.4
RIG-I	3.7	4.8	4.8	5.0
MAVS	7.5	8.8	8.6	8.4
IRF1	6.7	11.0	11.8	10.5
ISG15	6.1	8.0	8.8	8.2

## Data Availability

Data are contained within the article.
